# Enhancing the Antioxidant and Nutritional Profile of Gluten-Free Sourdough Bread Using Hemp Press Cake Meal

**DOI:** 10.3390/foods14203571

**Published:** 2025-10-20

**Authors:** Gjore Nakov, Marko Jukić, Francesca Soler, Semiha Syleimanova, Jasmina Lukinac, Lorenzo Estivi, Andrea Brandolini, Alyssa Hidalgo

**Affiliations:** 1 College of Sliven, Technical University of Sofia, Burgasko Shose Blvd 59A, 8800 Sliven, Bulgaria; gnakov@tu-sofia.bg; 2 Faculty of Food Technology Osijek, Josip Juraj Strossmayer, University of Osijek, F. Kuhaca 18, 31000 Osijek, Croatia; marko.jukic@ptfos.hr (M.J.); ptfosptfos2@gmail.com (J.L.); 3 Department of Food, Environmental and Nutritional Sciences (DeFENS), Università degli Studi di Milano, via Celoria 2, 20133 Milan, Italy; 4 Faculty of Engineering and Pedagogy, Technical University of Sofia, Burgasko Shose Blvd 59A, 8800 Sliven, Bulgaria; 5 Research Centre for Animal Production and Aquaculture (CREA-ZA), Council for Agricultural Research and Economics, Viale Piacenza 29, 26900 Lodi, Italy

**Keywords:** carotenoids, flavonoids, nutritional properties, phenolic acids, proteins, sensory evaluation, tocols

## Abstract

Food industries produce large amounts of by-products, valuable sources of bioactive compounds that can enhance gluten-free foods. This study evaluated the effect of hemp cake meal (HC) addition on the antioxidant, nutritional, technological, and sensorial properties of gluten-free sourdough breads. Rice flour, HC, and breads with increasing HC levels (0–30%) were analysed for ash, protein, lipids, soluble and insoluble fibre, phenols, tocols, and carotenoids. Technological traits, colour, and sensory quality were also assessed. HC showed higher contents of most compounds than rice flour. As a result, enriched breads displayed marked nutritional improvements: protein increased from 8.9 g/100 g (control) to 17.6 g/100 g (30% HC), lipids from 1.5 to 3.9 g/100 g, soluble fibre from 0.54 to 1.27 g/100 g, insoluble fibre from 3.2 to 13.4 g/100 g, and phenolics (mainly ellagic and rosmarinic acids, and caffeic and naringenin derivatives) from 10.8 to 174.8 mg/100 g. Tocols and carotenoids, though scarce, slightly increased. However, HC-enriched breads had lower volumes and a firmer, stickier texture. Nevertheless, up to 10% HC inclusion did not affect appearance, aroma, texture, or taste. Therefore, a moderate HC addition can improve antioxidants and the nutritional quality of gluten-free sourdough breads while maintaining a good sensory acceptability.

## 1. Introduction

The food industry produces millions of tons of by-products every year from various processes, often considered expensive waste to dispose of, despite being a potential source of low-cost bioactive compounds [1]. The growing global attention to waste reduction has fuelled interest in their reuse, spurring research into new uses for by-products or their derivatives in foods, pharmaceuticals, and cosmetics.

Hemp (*Cannabis sativa* L.) is a widely distributed crop, thriving in different climatic conditions. The different parts of the hemp plant are used by industries in the production of ropes, textiles, paper, bioplastics, cosmetics, essential oils, medicines, food additives, biofuels, and even in construction [2,3]. Hemp seeds are widely employed in the production of vegetable oil; its extraction generates large amounts of by-products, known as oil cakes. However, the current strategies to dispose of these oil cakes are associated with some shortcomings; therefore, identifying alternative approaches to reducing environmental impact and promoting a circular economy is a necessity [4].

The cake obtained after oil press extraction from the seeds, which can constitute up to 50% of the initial seed mass [5,6], still retains an excellent chemical composition, including 31.62% dry matter (DM) protein, 8.19% DM fat, and 43.76% DM dietary fibre [7], and is used mainly as animal feed [8]. However, because of its high protein content, it could be used to improve the nutritional qualities of existing products or to create new foods [9]. In recent years, the cake has been tested in the enrichment of several food products, such as wheat bread [7], pastries [9], ice cream [10], potato chips [11], etc.

Rice flour is one of the most widely used raw materials in gluten-free product development due to its favourable technological and nutritional characteristics. Compared to many alternative gluten-free flours, it has a neutral taste, white colour, and hypoallergenic profile, making it particularly suitable for consumers with celiac disease or gluten intolerance [12]. Its fine particle size and bland flavour facilitate the formulation of baked goods without introducing the off-flavours often associated with legumes or pseudocereals [13]. From a technological perspective, rice flour can produce breads with an acceptable texture and volume when properties such as amylose content, hydration, and water-holding capacity are optimised [14,15]. Nutritionally, rice flour is easily digestible and low in antinutritional factors, enhancing the bioavailability of nutrients [16]. These characteristics make rice flour a preferred base ingredient for gluten-free breads, pasta, and confectionery, and it frequently serves as a model flour in scientific studies [15].

The worldwide demand for gluten-free products has increased steadily, not only because the number of persons suffering from celiac disease is augmenting, but also because many people consider gluten-free products as a healthier food [17]. Celiac disease is a lifelong intestinal disease, with a prevalence of 0.7% to 1.4% in the general population, mainly triggered by gluten consumption, which can cause inflammation and swelling in the small intestine, preventing the absorption of essential nutrients such as calcium, iron, and fat-soluble vitamins [18]. The only cure for celiac disease is a complete and lifelong abstinence from foods containing gluten, the main constituent of the flour of several cereals (wheats, rye, triticale, barley, and possibly oats).

Gluten-free products often have a worse nutritional profile than those containing gluten, both in terms of macronutrients (due to a lack of protein) and micronutrients (due to a lack of B vitamins, iron, and calcium), and present marked sensory differences [19]; additionally, they have higher contents of carbohydrates, lipids, and sodium [20], leading to increased risks of diabetes, obesity, and cardiovascular disorders [21]. In the case of leavened products like bread, yeast somehow improves the overall nutritional quality of foods [22]. A further step in amending the nutritional balance is the utilisation of sourdough starters, which create a microbiologically complex environment where lactic acid bacteria (LAB) and yeast act simultaneously, providing nutritional and functional advantages compared to the use of baker’s yeast alone [23].

Recent studies have explored the application of sourdough fermentation in gluten-free products enriched with hemp or other seed flours. For example, Nissen et al. [24] reported that incorporating hemp seed flour in sourdough-fermented gluten-free products led to a more diverse profile of bioactive and antimicrobial volatile compounds, improved flavour retention, and enhanced functional properties compared to non-fermented controls. Similarly, Jagelaviciute and Cizeikiene [25] demonstrated that sourdoughs fermented with *Lactobacillus sanfranciscensis*, using hemp, chia, and quinoa flours, improved bread porosity, reduced staling, and achieved a higher sensory acceptability than breads prepared from non-fermented flours. These findings indicate that sourdough fermentation can effectively address the common limitations of gluten-free products, such as poor texture and rapid staling, while enhancing their nutritional and functional profile. Therefore, this approach represents a valuable strategy for improving the formulation and quality of gluten-free baked goods enriched with hemp or other nutrient-dense seed flours.

Some research exists in the literature on the addition of hemp cake to traditional or gluten-free breads [26,27,28]; however, these works have predominantly focused on technological changes, with a limited attention to nutritional and antioxidant properties. In line with European priorities for waste reduction, recycling, and valorisation of food by-products within a circular economy framework, as well as efforts to enhance sustainability in the food industry, the present study aims to comprehensively evaluate gluten-free sourdough rice bread enriched with 5%, 10%, 15%, 20%, 25%, or 30% hemp cake meal. Specifically, we investigate its chemical composition and antioxidant profile (including phenolic compounds, tocopherols, and carotenoids), as well as its physical, textural, and sensorial characteristics. This integrated approach provides novel insights into both the nutritional and structural effects of hemp cake meal enrichment in gluten-free sourdough bread.

## 2. Materials and Methods

### 2.1. Materials

For the production of gluten-free sourdough breads, the following ingredients were used: rice flour (Doves Farm, Hungerford, Berkshire, UK), water, and a commercial mixed starter culture (trade name LBB BR), produced by LB Bulgaricum, Sofia, Bulgaria. This starter culture contains a mixture of mesophilic and thermophilic strains, including *Lactobacillus brevis*, *Lactobacillus plantarum*, *Lactobacillus bulgaricus*, *Lactobacillus helveticus*, *Lactococcus lactis* ssp. *lactis*, *Lactococcus lactis* ssp. *cremoris*, *Lactococcus lactis* ssp. *lactis* var. *diacetylactis*, and *Leuconostoc mesenteroides* ssp. *cremoris*, unflavoured whey protein (Everbuild Whey Build, Tauranga, New Zealand), sugar (Zahira, Sofia, Bulgaria), salt (Izzi, Sofia, Bulgaria), dry yeast (Dr. Oetker, Bielefeld, Germany), guar gum (Zoya BG, Sofia, Bulgaria), sunflower oil (Tvornica ulja Čepin, Čepin, Croatia), corn starch (Dr. Oetker Kft, Janossomorja, Hungary) and hemp press cake meal, prepared under laboratory conditions.

### 2.2. Methods

#### 2.2.1. Production of Hemp Press Cake Meal (HC)

The meal was from hemp cake, a by-product of the oil extraction from hemp seeds of *Cannabis sativa* variety Finola, sourced from a local producer (Cannabio d.o.o., Sotin, Croatia). The oil extraction yield was 28%. The cake was ground with an IKA MF10 grinder (IKA^®^-Werke GmbH & Co. KG, Staufen, Germany) mill to flour <200 μm diameter. After milling, the meal was thoroughly dried to remove residual moisture before being used in formulations.

#### 2.2.2. Production of Gluten-Free Sourdough Bread

The sourdough was prepared by mixing 10 g rice flour, 20 mL water, and 0.25 g starter culture; the mixture was stored for 24 h at 25 °C. Separately, 90 g of rice flour (control) or a mixture of rice flour and HC (85:5; 80:10; 75:15; 70:20; 65:25, or 60:30), whey protein (5 g), corn starch (10 g), salt (1.8 g), sugar (2 g), and dry yeast (6 g) were thoroughly mixed for 2 min with a 1900 W spiral mixer (RL-PKM1900.7BG, Yangjiang, China). To these dry mixes, sourdough, sunflower oil (5 mL), xanthan gum (4 g), and water (100 mL) were added and kneaded for 10 min. All quantities are given on a 100 g flour basis. The loaves were produced using 200 g of flour each and weighed a total of 508.1 g. After kneading, the doughs were placed in a rectangular mould (12 × 8 × 5) and fermented for 45 min at 30 °C. Baking was performed in a preheated oven for 5 min at 200 °C, followed by 40 min at 175 °C (total: 45 min). Seven types of bread were produced: one control (0% HC) and six types with varying HC percentages (5%, 10%, 15%, 20%, 25%, and 30%). Bread samples were prepared in triplicate batches. All breads were cooled for 120 min at room temperature before being weighed and analysed to ensure consistent results.

#### 2.2.3. Chemical Analyses

The moisture and ash content of rice flour, hemp cake meal, and breads were determined according to methods 44-15.02 and 08-01.01 [29], respectively. The protein content was determined following a modified Lowry method [30]. The lipid content was determined as described in method ISO 6492:1999 [31]. The soluble dietary fibre (SDF), insoluble dietary fibre (IDF), and total dietary fibre (TDF) were determined through the enzymatic–gravimetric method using a dedicated Megazyme kit, following the AACC Method 32-05.01. Available carbohydrates were calculated by difference [100 − (moisture + ash + protein + fat + dietary fibre)]. The energy conversion factors applied were protein—17 kJ/g, fat—37 kJ/g, available carbohydrates—17 kJ/g, and dietary fibre—8 kJ/g, as reported by Menezes et al. [32]. All these analyses were performed in duplicate. The water activity (*A_w_*) was determined in triplicate at 25 °C with an Aqualab (Series 3 Quick Start, Decagon Device, Washington, DC, USA).

#### 2.2.4. Antioxidant Analysis

The content of the phenolic compounds, tocols, and carotenoids was determined for rice flour, HC, control bread, and the breads enriched with 10%, 20% and 30% HC. The analyses were structured into two main groups—phenolic compounds, and tocopherols and carotenoids—each described in detail in the following subsections.

#### 2.2.5. Phenolic Compounds

The analysis of the free soluble fraction of phenolic substances was performed using HPLC [33]. Briefly, 1.0 g of each raw material or bread sample was weighed in a capped centrifuge tube, and 15 mL of 80% *v*/*v* methanol–water solution was added. After stirring with a TX4 Digital IR Vortex Mixer (VELP Scientifica, Usmate Velate, Italy), the sample was placed in an FS200b ultrasonic bath (Decon, Hove, UK) for 5 min and in a PTR-35 shaker (Grant, Beaver Falls, PA, USA) for 30 min in a light-proof refrigerated environment. Subsequently, the sample was centrifuged at 15,000× *g* for 10 min at 8 °C in a LISA refrigerated centrifuge (AFI Groups, Chateau-Gontier, France). The supernatant was recovered in a 250 mL flask, and the extraction was repeated two more times by adding 15 mL to the remaining sediment. The extract was evaporated under vacuum at 35 °C for 30 min with a Laborota 4000 Efficient rotavapor (Heidolph, Schwabach, Germany) and brought to dryness with a nitrogen flow, then was resuspended with 2 mL methanol:water for chromatograph 80:20 (*v*/*v*), filtered on a 0.45 µm PTFE membrane (Diana Beck Scientific, Gallarate, Italy), and collected in an amber glass vial.

The chromatographic analysis was performed with a system composed of a Rheodyne injector with a 20 µL loop; L-2130 Elite LaChrom pump (VWR, Hitachi, Tokyo, Japan); Adamas C18 column, 250 mm × 4.6 mm, 5 µm and Adamas C18 guard column, 10 mm × 4.6 mm, 5 µm (Sepachrom SRL, Rho, Italy) thermostated at 30 °C with an L-2300 Elite LaChrom oven (VWR, Hitachi, Japan); Diode Array Detector L-2450 Elite LaChrom detector (Merck, Hitachi, Tokyo, Japan); and EZChrom Client/Server software (version 3.1.7). The elution was performed by a gradient of formic acid in water 99:1 *v*/*v* and acetonitrile at a flow rate of 1 mL/min. The different phenolic compounds were quantified at 280 nm, except caffeic acid, rosmarinic acid, and apigenin (320 nm). The quantification was performed using the external standard method with freshly prepared calibration curves. Compound identification was performed by comparing retention times and spectra of the standards; unidentified peaks were grouped based on absorbance similarity to the spectra of the standards and classified as their derivatives, in analogy to the previously published literature [34]. All the analyses were performed in duplicate, and the mean results are presented in mg/kg DM.

#### 2.2.6. Tocopherols and Carotenoids

Tocol and carotenoid extracts were obtained from all samples after thermal saponification [34]. Briefly, 1 g of each raw material or bread sample was weighed into a screw-capped glass tube and stirred with a TX4 Digital IR Vortex Mixer (VELP Scientifica, Usmate Velate, Italy) upon the addition of each of the following reagents: 2.5 mL 60 g/L pyrogallolic ethanol, 1 mL 95% ethanol, 1 mL 10 g/L sodium chloride, and 1 mL 600 g/L potassium hydroxide. After blowing nitrogen into the tube, saponification was performed at 70 °C for 45 min, under darkness. During the saponification, the tubes were vortexed every 10 min. At the end, they were cooled in an ice bath; 7.5 mL of 10 g/L sodium chloride was added, and the mixture was transferred into 250 mL flasks, where it underwent liquid–liquid extraction twice by stirring with 15 mL of n-hexane:ethyl acetate 90:10 (*v*/*v*) before being centrifuged at 15,000× *g* for 10 min at 8 °C. The supernatants were pooled in a 250 mL flask, and the solvent was removed under vacuum in a Laborota 4000 Efficient rotavapor (Heidolph, Schwabach, Germany) at 35 °C for 5 min. The extracts were dried under nitrogen flow for 1 min, resuspended with 2 mL of n-hexane:2-propanol 99:1 (*v*/*v*), and filtered through a 0.45 µm PTFE membrane (Diana Beck Scientific, Gallarate, Italy). The chromatographic analysis was performed by normal-phase HPLC [35] in a system comprising the following: Rheodyne injector with 50 µL loop; Adamas^®^ Silica column 250 mm × 4.6 mm, 5 μm, non-thermostatted, equipped with a 10 × 4.6 mm, 5 μm pre-column (Sepachrom SRL, Rho, Italy); L-2130 Elite LaChrom pump (VWR, Hitachi, Tokyo, Japan); and 821 FP fluorimetric detector (Jasco, Tokyo, Japan), set at excitation wavelengths of 290 nm and emission wavelengths of 330 nm, managed by Empower 2 software (Waters Chromatography Division, Millipore, Milford, CT, USA) via the SAT/IN interface (Waters). As the mobile phase, a mixture of n-hexane:ethyl acetate:acetic acid 97.3:1.8:0.9 (*v*/*v*/*v*), with a flow rate of 1.6 mL/min, was used. The peaks were quantified using external standards of α-tocopherol, β-tocopherol, γ-tocopherol, and δ-tocopherol, while the tocotrienols were quantified using the standard curves of the corresponding tocopherols.

For the quantification of carotenoids, the same instrumentation was used but with a PDA 2996 detector (Waters Chromatography Division, Millipore, Milford) at a wavelength of 445 nm, and a mixture of n-hexane:2-propanol 95:5 (*v*/*v*) was used as the mobile phase, with a flow rate of 1.5 mL/min. Peak quantification was performed using the external standard method. All the analyses were performed in duplicate. The mean results are presented as mg/kg DM.

#### 2.2.7. Physical, Colour, Textural Characteristics and Image Analysis

The volume (cm^3^) of the bread with different quantity of HC was determined with a Volscan Profiler (Stable Micro Systems Ltd. Godalming, Surrey, UK). The colour of the crust and crumb was determined with a Chroma Meter CR-400 colorimeter (Konica Minolta, Tokyo, Japan) on two sets of five random breads. Texture profile analysis (TPA) of the bread samples was carried out using a TA.XTplus analyzer (Stable Micro Systems Ltd., Surrey, UK). The loaves were sliced into uniform pieces 2.5 cm thick, and two central slices were selected for double compression (40% slice thickness) using a 36 mm diameter cylindrical aluminium probe. Force–time curves were recorded at a crosshead speed of 1.7 mm/s, with a trigger force of 0.049 N, and the resting time between two compressions was 5 s. From these curves, textural parameters such as hardness, springiness, cohesiveness, chewiness, resilience, and adhesiveness were determined. Each measurement was performed on six slices (three loaves from different batches), and the results are expressed as mean ± standard deviation. Computer image analysis was used to quantify the crumb structure of bread, providing objective data on porosity and cavity distribution [36]. Bread slices were scanned using an EPSON Perfection V500 Photo scanner (800 dpi, 24-bit RGB, TIFF format). Images were processed in ImageJ v1.54g software(ImageJ, National Institutes of Health, Bethesda, MD, USA), where the central crumb region was selected as the region of interest (ROI), converted to 8-bit grayscale, and segmented. Segmented images were analysed. The following parameters were calculated for each sample: pore count (n/cm^2^), average pore size (mm^2^) and porosity (%).

The sensory analysis of the seven different types of gluten-free sourdough breads was performed at University of Osijek, Faculty of Food Technology, Croatia. This research was approved by the Ethics Committee of the Faculty of Food Technology at Josip Juraj Strossmayer University of Osijek, Croatia (Class number: 602-04/24-08/01). Twenty semi-trained people participated in the sensory analysis, after providing an informed consent according to the guidelines for Ethics and Food-Related research defined by the European Union [37]. All panellists had prior experience with sensory analysis and received a short orientation session prior to testing, during which the evaluated attributes were defined and explained, and example samples were provided to ensure a common understanding of the terminology. The evaluation was conducted in a sensory laboratory under controlled conditions (daylight-type illumination, 22 ± 2 °C, individual booths). Bread loaves were baked on the day of testing and cooled to room temperature before being cut into uniform 2 cm slices. Samples were served on identical white plastic plates, coded with random three-digit numbers to ensure blind evaluation. The serving order was randomised across panellists to minimise order effects. Still water and unsalted crackers were provided for palate cleansing between samples. The evaluation followed a structured 5-point descriptive intensity scale, assessing appearance, texture, aroma, taste, and overall acceptability (1 = extreme dislike, 5 = extreme like). Each attribute was explained to the panellists prior to testing:

- Appearance: colour of crust and crumb, uniformity, visual appeal;
- Texture: crumb structure, softness, elasticity, mouthfeel;
- Aroma: intensity and pleasantness of bread aroma, presence of hemp-related notes;
- Taste: flavour balance, intensity, presence of off-flavours;
- Overall acceptability: general impression and likelihood of consumption.

All panellists independently scored each sample. Data were analysed using analysis of variance (ANOVA) followed by Tukey’s HSD post hoc test (*p* < 0.05) to determine significant differences among formulations. Results are presented as mean values ± standard deviation. It should be emphasised that this assessment was conducted as a semi-trained panel evaluation, intended to provide comparative and indicative differences among the breads enriched with HC, rather than as a large-scale consumer hedonic test.

#### 2.2.8. Statistical Analysis

A one-way analysis of variance (ANOVA) was conducted, and, when significant differences were found, Fisher’s Least Significant Difference (LSD) test was applied at a significance level of *p* < 0.05. The analyses were carried out using the software XL STAT 2019 (Addinsoft Inc., Long Island City, NY, USA) and Microsoft Office Excel 2019 (Microsoft, Redmond, WA, USA).

## 3. Results and Discussion

### 3.1. Chemical Composition of Rice Flour and Hemp Cake Meal

**Figure 1:**
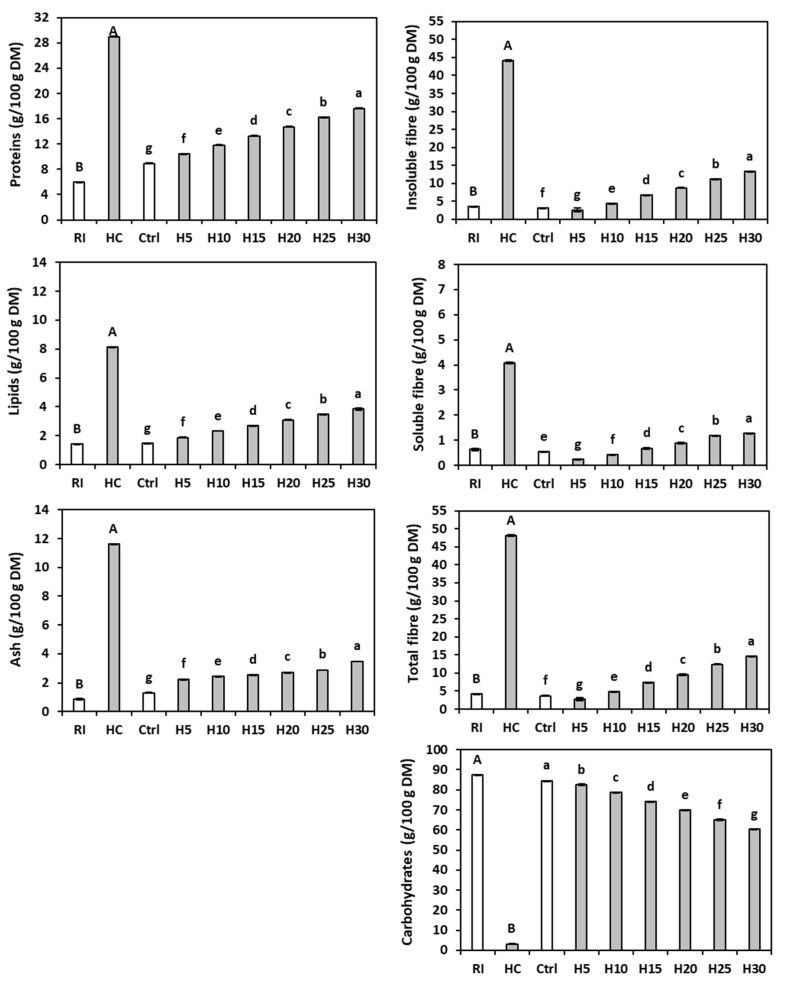
Chemical composition of rice (RI), hemp cake meal (HC), gluten-free control bread (Ctrl), and gluten-free breads enriched with 5% (H5), 10% (H10), 15% (H15), 20% (H20), 25% (H25), or 30% (H30) hemp cake meal. The bars represent the standard deviation. Different letters indicate statistical differences (*p* < 0.05) between flours (upper case) or among breads (lower case) following Fisher’s LSD test.

The analysis of variance revealed significant differences between rice flour and hemp cake meal for all compounds tested. The moisture content of rice flour (8.84 ± 0.00 g/100 g) was significantly (*p* < 0.05) superior to that of hemp cake meal (6.80 ± 0.66 g/100 g). A higher protein content in hemp cake meal (29.00 ± 0.07 g/100 g DM) compared to the rice flour (5.98 ± 0.04 g/100 g DM) was also found (Figure 1). The hemp cake meal protein value was within the ranges (23.25–33.45 and 24.8–36.1 g/100 g DM) reported [38,39], while the rice content was slightly lower than the results (7.29 ± 0.02 and 7.57 g/100 g DM) described by some authors [40,41], but was similar to that (5.55 g/100 g DM) stated by Ren et al. [42]. The small differences may be due to the cropping environment [43], the degree of milling [44], and/or the storage conditions [45].

The lipid content of the hemp cake meal (8.13 ± 0.04 g/100 g DM) was significantly higher than that of rice flour (1.42 ± 0.02 g/100 g DM) and agrees with the results (8.37 ± 1.17 g/100 g DM) reported by Mohamed et al. [46], as well as the range (8.9–16.4 g/100 g DM) observed by Rakita et al. [39].

The rice flour also had a much lower ash content (0.87 ± 0.00 g/100 g DM) than the hemp cake meal (11.60 ± 0.02 g/100 g DM). A lower ash concentration in hemp cake meal (6.86 g/100 g DM) was described [47], probably due to the differences in variety [48], agronomic and environmental conditions [49], and/or oil extraction parameters [50]. According to Mohamed et al. [51], HC ash contains phosphorus, potassium, magnesium, iron, and manganese.

Not surprisingly, the refined rice flour contained significantly less soluble fibre (0.63 ± 0.04 g/100 g DM), insoluble fibre (3.55 ± 0.07 g/100 g DM), and total fibre (4.18 ± 0.04 g/100 g DM) than the hemp cake meal (4.07 ± 0.03, 44.09 ± 0.28 and 48.16 ± 0.25 g/100 g DM, respectively). However, a predominance of the insoluble fraction over the soluble one was evident in both raw materials. The total fibre content in the hemp cake was similar to previously reported results (46.14 ± 0.09 g/100 g DM) [52]. Conversely, the rice flour had the highest available carbohydrate content (87.56 ± 0.02 g/100 g DM), with values similar to known levels (90 and 89–91 g/100 g DM) [53,54], while the hemp cake meal showed significantly lower levels (3.12 ± 0.12 g/100 g DM).

### 3.2. Chemical Composition of Breads

The analysis of variance revealed significant differences between bread samples for almost all the compounds analysed. The addition of the hemp by-product led to an increase in moisture from 39.0 ± 0.6 g/100 g (control bread) to between 42.7 ± 1.0 g/100 g (H5) and 45.5 ± 0.1 g/100 g (H30). Dietary fibres are well known for their high water-holding capacity, which can significantly influence the moisture content of baked goods. By binding free water in the dough matrix, the fibres limit water mobility during baking and reduce water evaporation during baking, leading to a higher residual moisture in the final product [55,56].

Water activity is a key parameter for food safety, quality, shelf life, and microbial stability because it affects microbial growth more than moisture content [57]. The *A_w_* range of variation (0.84–0.89) among breads was minimal and the differences not significant.

The HC addition increased the protein and the lipid contents according to the percentage of by-product present (Figure 1), and the lipid concentrations of H10 and H20 were similar to those reported by Korus et al. [26]. Instead, the ash content of the enriched breads was higher than that of the control bread but did not follow a trend proportional to the level of addition. The total fibre content of the breads with hemp cake meal was significantly higher than that of the control (3.69 ± 0.09 g/100 g DM), with an increase proportional to the by-product percentage, reaching 14.62 ± 0.09 g/100 g DM in H30.

As stated, HC is richer in ash, protein, fat, and dietary fibre compared to rice flour, and therefore an increase in HC percentage fosters their content in the enriched breads [7,58,59]. On the other hand, the available carbohydrate content of the control (84.61 ± 0.01 g/100 g DM), primarily from rice flour, was significantly higher than that of the enriched breads; therefore, the carbohydrates content decreased as the percentage of raw material in the dough increased.

### 3.3. Phenolic Content of Rice Flour and Hemp Cake Meal

**Figure 2:**
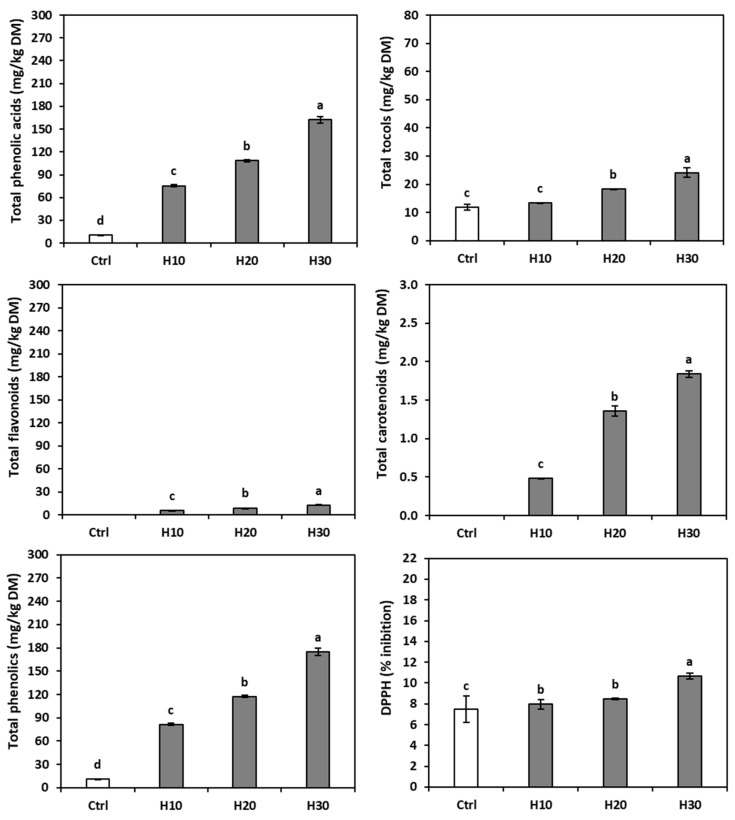
Antioxidants content (mg/kg DM) and antioxidant capacity (DPPH) of gluten-free sourdough control bread (Ctrl) and gluten-free sourdough breads enriched with 10% (H10), 20% (H20), or 30% (H30) hemp cake meal. The bars represent the standard deviation. Different letters indicate statistical differences (*p* < 0.05) between flours (upper case) or among breads (lower case) following Fisher’s LSD test.

The ANOVA (not presented) demonstrated that the raw materials showed significant differences in total phenolic acid and total flavonoid contents. Table 1 reports the content of individual phenolic acids and flavonoids in the raw materials and breads, while Figure 2 depicts their total content. The rice flour contained only ferulic and *p*-coumaric acids, as already noticed [60,61]; instead, nine compounds (the phenolic acids caffeic, caffeic derivative, chlorogenic, ellagic derivative, protocatechuic, rosmarinic, and syringic, and the flavonoids apigenin derivative and naringenin derivative) were identified in the hemp cake meal. The total phenolic acids were more abundant than the total flavonoids (467.36 ± 9.08 and 39.41 ± 1.33 mg/kg DM, respectively). Among the phenolic acids observed by other authors are protocatechuic, chlorogenic, caffeic, and syringic acids and naringenin [62]; ellagic acid and apigenin [63]; syringic acid, caffeic acid, naringenin, and apigenin [64]; and caffeic, chlorogenic, and protocatechuic acids [65,66] and syringic acid [67].

In the control bread, only ferulic, *p*-coumaric, *p*-coumaric derivative, and vanillic acids were detected (Table 1), the first two coming from the rice flour and the latter two from the sunflower oil in the formulation [68], for a total of 10.81 ± 0.06 mg/kg DM phenolic content (Figure 2). In the HC-enriched breads, the ferulic, *p*-coumaric, and *p*-coumaric derivative acids showed amounts similar to those observed in the control bread, while the vanillic acid was below the detection limit. All the phenolic compounds from the hemp cake meal increased in the breads as a function of the enrichment percentage. However, the results also suggested that caffeic, protocatechuic, and syringic acids were more sensitive to the process, while caffeic derivative, chlorogenic, and apigenin derivative acids were more stable. Ellagic acid, rosmarinic acid, and naringenin derivative exhibited higher concentrations than expected, indicating the greater extractability of the compounds. Overall, in the breads formulated with the hemp by-products, the phenol content increased according to the percentage of addition.

### 3.4. Phenolic Content of the Breads

#### 3.4.1. Tocol and Carotenoid Content of Rice Flour and Hemp Cake Meal

Table 1 shows the tocol and carotenoid composition and content of both the raw materials and the enriched breads. The rice flour contained only small amounts of β-tocopherol and γ-tocotrienol, not present in the by-product, while the hemp cake meal had a sizeable content of γ-tocopherol and smaller amounts of α-tocopherol and δ-tocopherol. The presence of β-tocopherol and γ-tocotrienol in rice was already reported [69], and α-tocopherol, γ-tocopherol, and δ-tocopherol were spotted in hemp cake meal [63,70], although only α-tocopherol and γ-tocopherol were found by Siano et al. [71]. The overall total tocol content in hemp cake meal (91.49 ± 4.01 mg/kg DM) was approximately nine times higher than that in rice flour (11.28 ± 0.31 mg/kg DM).

The carotenoids were absent in the rice flour but were present in the hemp cake meal (14.45 ± 0.33 mg/kg DM); the most abundant compound was lutein (13.72 ± 0.31 mg/kg DM), associated with traces of β-carotene, β-cryptoxanthin, and zeaxanthin. The presence of lutein and β-carotene in HC was described by [72,73].

#### 3.4.2. Tocol and Carotenoid Content of the Breads

The control bread (Table 1) contained small amounts of β-tocopherol and γ-tocotrienol, evidently derived from the rice flour, as well as α-tocopherol, likely from the sunflower oil in the formulation. Indeed, Wen et al. [74] reported 542.1–870.5 mg/kg in this oil, and Arias-Santé et al. [75] and Pointner et al. [76] reached similar conclusions. The α-tocopherol and γ-tocopherol levels of the hemp-enriched breads increased according to the hemp flour percentage, while the γ-T3 was slightly lower than in the control. On the other hand, the β-tocopherol content was similar across breads, and the δ-T, already scarce in the HC, was undetectable in the breads. Therefore, the breads with HC contained more total tocols than the control bread (Figure 2).

The lutein content increased progressively in the enriched breads but was still scarce. The zeaxanthin was barely detectable in the breads with HC, while the β-carotene and the β-cryptoxanthin dipped below the detection limit. Overall, the total carotenoid content in the enriched breads was higher than in the control and increased with increasing by-product amounts (Figure 2).

### 3.5. Bread Colour

The addition of hemp cake (HC) significantly influenced the colour parameters of both the crust and the crumb (Table 2). The L* values decreased progressively with increasing HC incorporation in both bread fractions, reflecting the darker intrinsic colour of HC and its diluting effect on the light rice flour matrix.

In the crust, colour changes were dominated by thermal reactions. Intense Maillard and caramelisation processes generated melanoidins that deepened brown shades and reduced lightness, while simultaneously decreasing chroma (C*), as pigments were degraded. The a* values decreased (8.7 → 4.5) with increasing HC, indicating a loss of reddish tones and a shift towards green-brown hues. This is attributable both to the thermal breakdown of red-brown pigments and to the presence of chlorophylls and phenolics from HC, which, upon heating, are transformed into derivatives with a dull green-brown appearance [77]. In parallel, b* values also declined (31.7 → 22.7), although they remained relatively high, consistent with a persistent yellow component. The rise in hue angle (*h°* 74.7 → 78.9) confirmed a perceptible shift from reddish-yellow to more yellow-green tones. These patterns are in line with previous studies reporting a reduced redness and chroma in gluten-free breads enriched with hemp ingredients [26,78].

In the crumb, although L* values also decreased (75.4 → 43.2), both a* and b* values significantly increased (*p* < 0.05). Despite their low absolute magnitude (a* from 0.2 to 1.5), this trend reflects a shift from an almost achromatic white crumb towards warmer tones. The rise in a*, along with b*, can be attributed to preserved, non-thermalised hemp pigments and mildly developed Maillard intermediates that imparted subtle yellow-orange hues. The decrease in *h°* (89.0 → 85.5) corroborates this interpretation, as it denotes a shift from pure yellow towards orange. Simultaneously, chroma values increased (C* 13.9 → 19.5), indicating a higher saturation, particularly within the yellow–red domain.

The divergent direction of a* between crust and crumb thus reflects differences in the thermal regime: the crust is subjected to a strong heating, favouring pigment degradation and the dominance of brown Maillard polymers, whereas the crumb retains partially intact hemp-derived pigments and moderately altered colour compounds. Similar phenomena have been described for seed- and fibre-enriched breads, where the crust and crumb exhibited distinct colour trajectories depending on the stability of incorporated pigments and the severity of thermal processing [26,78,79].

In addition to the influence of HC pigments and thermal load, sourdough fermentation also contributed to colour development. Organic acids such as lactic and acetic acid lower the pH of the dough, which can modulate the course of Maillard reactions and influence pigment stability. While a lower pH may not universally accelerate Maillard browning, sourdough fermentation alters both the precursor availability (free amino acids, reducing sugars) and pH conditions, creating a combined effect that enhances the overall browning intensity in the crust [79,80].

Overall, these findings suggest that HC supplementation drives a consistent darkening of both the crust and crumb (L* decrease), but with divergent chromatic shifts: the crust loses redness and chroma due to pigment degradation and thermal browning, while the crumb develops warmer and more saturated tones due to pigment preservation and moderate browning.

### 3.6. Technological Quality of the Breads

Table 3 shows the physical characteristics of gluten-free sourdough breads enriched with hemp cake meal. The incorporation of starter cultures in gluten-free bread formulations has a significant impact on both the technological and nutritional properties, thereby improving the overall quality of the final product. Starter cultures, particularly those containing lactic acid bacteria (LAB) and yeasts, contribute to better dough rheology, texture, and loaf volume by producing organic acids such as lactic and acetic acids. These acids lower the pH, which strengthens the dough structure and enhances leavening. Additionally, the acidification process helps extend shelf life by inhibiting spoilage microorganisms. From a nutritional standpoint, LAB fermentation can improve mineral bioavailability by reducing antinutritional factors such as phytic acid, enhance protein digestibility, and generate bioactive peptides and essential amino acids, all of which enhance the nutritional profile of the bread. Several studies have demonstrated these effects: Keramari et al. [81] showed that commercial starter cultures improved both the technological and nutritional quality of gluten-free rice/chickpea sourdough bread; Kulathunga et al. [82] reported a higher protein digestibility in sourdoughs fermented with rye starter cultures compared to wheat-based starters; and Atfaouri et al. [83] highlighted that starter culture selection plays a crucial role in leavening, bio-preservative potential, and overall bread quality.

The volume of the loaves decreased significantly (*p* < 0.05) with increasing hemp content, as noticed also by other authors [84,85]. The hemp cake meal is rich in dietary fibre, and especially insoluble fibre, which can inhibit dough expansion and also limits CO_2_ retention during fermentation [86]. Furthermore, the fibre has a high water binding capacity, reducing the water available for the complete hydration of the dough and limiting its expansion during baking [7]. However, a volume increase in gluten-free bread enriched with HC was observed and attributed it to the surfactant and foaming properties of hemp protein, which contribute to the stabilisation of CO_2_ in the bread [26].

The hardness increased from 17.1 N in the control bread to 26.5 N in the bread with 30% hemp cake meal, in concordance with the results obtained by Capcanacari et al. [7] and Marinoupolou et al. [7,84]. The abundant insoluble dietary fibre and proteins preferentially absorb water, preventing starch gelatinization and air cell formation [87]. The adhesiveness also increased from 0.11 N/s to 0.33 N/s with increasing HC percentages. Probably, the high water binding capacity and the presence of soluble dietary fibres create a wetter environment, increasing the stickiness of the breads [84].

On the contrary, the elasticity decreased with increasing hemp cake meal content, losing the ability to return to its original shape after compression [88]. The cohesiveness of the breads did not change significantly, diminishing only slightly from 0.63 in the control bread to 0.61 in the bread with 30% hemp cake meal. On the contrary, a certain decrease in cohesiveness with increasing hemp flour content is reported [84], possibly because the fibres disrupt starch gel formation, making the mixture more prone to disintegration and structural failure under pressure [86]. Finally, the chewiness increased significantly (*p* < 0.05), passing from 9.8 in the control up to 16.9 in the bread with 30% hemp cake meal as a result of the higher resistance to deformation, because the protein–fibre matrix enriched with hemp cake meal does not allow proper expansion, therefore increasing the mechanical effort required for chewing. Similar results were presented by Marinopoulou et al. [84].

**Figure 3:**
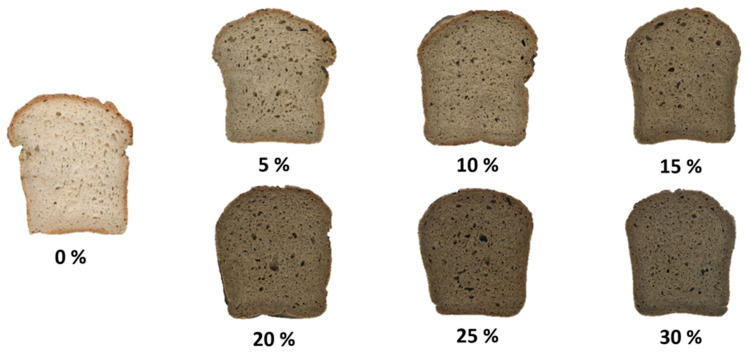
Cross-section of bread samples enriched with hemp cake meal.

A lower loaf volume and firmer texture can be directly linked to an altered bread cell structure (Figure 3), as measured through digital image analysis (Table 4). Although the overall porosity percentage remained statistically unchanged (20–23%), significant changes in pore distribution were observed. Increasing the addition of hemp cake meal resulted in a higher pore count per unit area (from 12.8 n/cm^2^ in the control to 26.6 n/cm^2^ for the 30% hemp cake meal), while simultaneously and significantly reducing the average pore size (from 1.20 mm^2^ to 0.86 mm^2^). This indicates that breads with higher hemp cake meal content developed a finer but denser crumb structure. Such crumb compactness accounts for the observed decrease in bread volume and the increase in hardness and chewiness, as smaller pores produce less compressible crumb walls. These findings may be explained by the presence of hemp proteins, which provide some degree of structural continuity, though not enough to offset the loss of gas-holding capacity caused by fibre enrichment. Our results are consistent with previous image analysis studies showing that hemp enrichment leads to an increased pore density and reduced average pore size, which correlate with a decreased bread volume and increased hardness [89].

Furthermore, in addition to the effect of dietary fibre, changes in crumb structure associated with the inclusion of hemp flour (reduced volume, smaller pores, and a denser matrix) may further enhance water retention. From the perspective of heat and mass transport phenomena in porous media, the denser crumb structure limits the pathways for heat transfer and vapour diffusion, which delays the temperature rise in the crumb during baking and slows moisture migration and evaporation [90,91].

### 3.7. Sensorial Analysis of the Breads

Supplementary Figure S1 presents the visual texture of rice flour, hemp cake, and gluten-free sourdough breads. The sensory analysis showed that the addition of up to 10% hemp flour did not significantly change the panel scores of appearance, texture, aroma, taste, and overall acceptance (Table 5) when compared to the control, while the acceptability of the other breads decreased rapidly with increasing HC percentages.

Our results bridge those described by Hayward et al. [92], who observed the best sensory qualities in gluten-free bread containing 5% hemp cake meal, and by Capnacari et al. [7], who showed that bread with 30% and 40% hemp cake meal is more difficult to accept for consumers. This trend may be partially due to the well-known lower acceptability of breads (normal and gluten-free) prepared with the contribution of different whole meals [93]. Nevertheless, the good sensorial performance of our gluten-free breads with up to 10% hemp cake meal, coupled with the better nutritional composition and antioxidant content, suggests that they may easily find a place in the diet of celiac people and also in the daily consumption of non-celiac customers.

## Figures and Tables

**Table 1:** Phenolic acids, flavonoids, tocols, and carotenoids (mg/kg DM ± standard deviation) in rice flour (RI), hemp cake meal (HC), gluten-free sourdough control bread (Ctrl), and gluten-free sourdough breads enriched with 10% (H10), 20% (H20), or 30% (H30) hemp cake meal. Different letters indicate statistical differences (*p* < 0.05) among breads following Fisher’s LSD test.

	RI	HC	Control	H10	H20	H30
*Phenolic acids*						
Caffeic		3.77 ± 0.34		0.26 ^c^ ± 0.02	0.62 ^b^ ± 0.01	1.19 ^a^ ± 0.08
Caffeic der.		132.4 ± 3.6		13.98 ^c^ ± 0.57	20.99 ^b^ ± 0.17	33.20 ^a^ ± 1.75
Chlorogenic		29.78 ± 0.47		3.89 ^c^ ± 0.01	6.80 ^b^ ± 0.11	10.10 ^a^ ± 0.15
Ellagic der.		83.30 ± 0.78		13.75 ^c^ ± 0.41	21.50 ^b^ ± 0.19	33.15 ^a^ ± 0.06
Ferulic	3.50 ± 0.26		3.30 ^b^ ± 0.03	4.15 ^a^ ± 0.14	3.79 ^ab^ ± 0.11	3.66 ^ab^ ± 0.04
*p*-coumaric	0.89 ± 0.12		2.27 ^b^ ± 0.11	3.28 ^a^ ± 0.06	3.33 ^a^ ± 0.00	3.54 ^a^ ± 0.04
*p*-coumaric der.			2.86 ^a^ ± 0.29	3.04 ^a^ ± 0.04	2.51 ^ab^ ± 0.02	2.45 ^b^ ± 0.00
Protocatechuic		79.62 ± 3.80		3.72 ^c^ ± 0.21	4.05 ^b^ ± 0.02	5.95 ^a^ ± 0.11
Rosmarinic		170.9 ± 6.3		27.21 ^c^ ± 2.16	42.47 ^b^ ± 1.08	65.02 ^a^ ± 2.62
Syringic		33.95 ± 0.06		2.13 ^c^ ± 0.04	2.39 ^b^ ± 0.03	4.01 ^a^ ± 0.08
Vanillic			2.39 ± 0.22			
*Flavonoids*						
Apigenin der.		6.78 ^±^ 0.49		0.64 ^b^ ± 0.03	1.05 ^a^ ± 0.00	1.29 ^a^ ± 0.08
Naringenin der.		32.63 ± 1.82			7.87 ^b^ ± 0.01	11.24 ^a^ ± 0.48
*Tocols*						
α-T		7.38 ± 0.58	3.68 ^c^ ± 0.39	4.69 ^b^ ± 0.40	5.24 ^a^ ± 0.05	6.45 ^a^ ± 0.55
β-T	4.84 ± 0.38		3.73 ^a^ ± 0.27	4.53 ^a^ ± 0.40	3.92 ^a^ ± 0.30	3.67 ^a^ ± 0.47
γ-T		81.66 ± 3.37		2.40 ^c^ ± 0.07	7.14 ^b^ ± 0.02	11.79 ^a^ ± 0.48
γ-T3	6.44 ± 0.07		4.48 ^a^ ± 0.31	1.75 ^c^ ± 0.07	2.02 ^b^ ± 0.19	2.20 ^b^ ± 0.15
δ-T		2.45 ± 0.05				
*Carotenoids*						
β-carotene		0.26 ± 0.02				
β-cryptoxanthin		0.15 ± 0.03				
Lutein		13.72 ± 0.31		0.46 ^c^ ± 0.01	1.32 ^b^ ± 0.06	1.80 ^a^ ± 0.05
Zeaxanthin		0.15 ± 0.00		0.03 ± 0.00	0.04 ± 0.01	0.03 ± 0.00

der.: derivative.

**Table 2:** Colour parameters (L*, a*, b*, C*, and *h*°, mean ± standard deviation) of crust and crumb of gluten-free control bread and gluten-free breads enriched with 5% (H5), 10% (H10), 15% (H15), 20% (H20), 25% (H25), or 30% (H30) hemp cake meal. Values in the same column with different letters are statistically different (*p* < 0.05) following Fisher’s LSD test.

Sample		L*	a*	b*	*C*	*h*
Crust	Control	60.1 ± 3.0 ^a^	8.7 ± 0.8 ^a^	31.7 ± 1.4 ^a^	32.9 ± 1.5 ^a^	74.7 ± 0.9 ^c^
	H5	54.0 ± 1.6 ^b^	6.8 ± 0.4 ^b^	28.5 ± 1.0 ^b^	29.3 ± 1.0 ^b^	76.6 ± 0.9 ^b^
	H10	50.4 ± 1.9 ^bc^	5.9 ± 0.5 ^c^	27.0 ± 1.1 ^bc^	27.6 ± 1.1 ^c^	77.7 ± 1.2 ^ab^
	H15	46.9 ± 3.7 ^cd^	5.4 ± 0.7 ^cd^	25.4 ± 1.2 ^cd^	25.9 ± 1.2 ^d^	78.0 ± 1.7 ^ab^
	H20	44.9 ± 2.2 ^de^	5.0 ± 0.3 ^de^	25.0 ± 1.2 ^d^	25.5 ± 1.2 ^d^	78.7 ± 0.8 ^a^
	H25	42.7 ± 2.0 ^ef^	4.6 ± 0.7 ^de^	23.3 ± 0.8 ^e^	23.8 ± 0.7 ^e^	78.7 ± 1.9 ^a^
	H30	39.9 ± 3.2 ^f^	4.5 ± 0.5 ^e^	22.7 ± 1.3 ^e^	23.2 ± 1.3 ^e^	78.9 ± 1.3 ^a^
Crumb	Control	75.4 ± 1.2 ^a^	0.2 ± 0.1 ^c^	13.9 ± 0.5 ^d^	13.9 ± 0.5 ^d^	89.0 ± 0.4 ^a^
	H5	61.8 ± 1.9 ^b^	0.3 ± 0.2 ^c^	18.0 ± 0.3 ^c^	18.0 ± 0.3 ^c^	89.0 ± 0.6 ^a^
	H10	55.9 ± 1.0 ^c^	0.7 ± 0.2 ^b^	18.6 ± 0.8 ^bc^	18.6 ± 0.8 ^bc^	87.9 ± 0.5 ^b^
	H15	49.7 ± 1.2 ^d^	1.3 ± 0.1 ^a^	18.7 ± 0.5 ^bc^	18.7 ± 0.5 ^b^	86.0 ± 0.4 ^c^
	H20	47.3 ± 0.7 ^e^	1.3 ± 0.1 ^a^	18.8 ± 0.7 ^ab^	18.9 ± 0.7 ^ab^	86.0 ± 0.4 ^c^
	H25	45.0 ± 1.6 ^f^	1.5 ± 0.2 ^a^	19.0 ± 0.4 ^ab^	19.1 ± 0.4 ^ab^	85.6 ± 0.7 ^c^
	H30	43.2 ± 1.6 ^f^	1.5 ± 0.2 ^a^	19.5 ± 0.3 ^a^	19.5 ± 0.3 ^a^	85.5 ± 0.5 ^c^

**Table 3:** Physical characteristics (mean ± standard deviation) of gluten-free sourdough control bread (Ctrl) and of gluten-free sourdough breads enriched with 5% (H5), 10% (H10), 15% (H15), 20% (H20), 25% (H25), or 30% (H30) hemp cake meal. Values in the same column with different letters are statistically different (*p* < 0.05) following Fisher’s LSD test.

	Volume (cm^3^)	Hardness (N)	Adhesiveness (N/s)	Springiness	Cohesiveness	Chewiness (N)
Control	622.1 ± 6.2 ^a^	17.1 ± 1.4 ^d^	0.11 ± 0.01 ^e^	0.89 ± 0.01 ^bc^	0.63 ± 0.02 ^a^	9.8 ± 1.1 ^e^
H5	569.1 ± 2.2 ^b^	18.5 ± 1.1 ^d^	0.19 ± 0.04 ^d^	0.90 ± 0.01 ^ab^	0.66 ± 0.02 ^a^	11.4 ± 1.6 ^de^
H10	556.2 ± 7.9 ^bc^	19.7 ± 1.4 ^cd^	0.24 ± 0.01 ^cd^	0.90 ± 0.00 ^abc^	0.65 ± 0.02 ^a^	12.5 ± 1.7 ^cd^
H15	544.8 ± 19.9 ^bc^	21.8 ± 1.2 ^bc^	0.26 ± 0.03 ^bcd^	0.91 ± 0.00 ^a^	0.67 ± 0.03 ^a^	13.5 ± 0.3 ^bcd^
H20	537.4 ± 12.8 ^bc^	23.9 ± 1.0 ^ab^	0.29 ± 0.03 ^abc^	0.88 ± 0.01 ^c^	0.63 ± 0.03 ^a^	14.7 ± 0.4 ^abc^
H25	527.8 ± 1.7 ^c^	25.9 ± 1.3 ^a^	0.31 ± 0.01 ^ab^	0.88 ± 0.00 ^c^	0.63 ± 0.01 ^a^	15.8 ± 1.3 ^ab^
H30	526.6 ± 5.1 ^c^	26.5 ± 1.2 ^a^	0.33 ± 0.02 ^a^	0.88 ± 0.01 ^c^	0.61 ± 0.04 ^a^	16.9 ± 0.5 ^a^

**Table 4:** Bread cell structure (mean ± standard deviation) of gluten-free sourdough control bread (Ctrl) and gluten-free sourdough breads enriched with 5% (H5), 10% (H10), 15% (H15), 20% (H20), 25% (H25), or 30% (H30) hemp cake meal. Values in the same column with different letters are statistically different (*p* < 0.05) following Fisher’s LSD test.

Sample	Pore Count (n/cm^2^)	Average Pore Size (mm^2^)	Porosity (%)
Control	12.78 ± 2.20 ^c^	1.20 ± 0.14 ^a^	20.62 ± 2.69 ^a^
H5	16.03 ± 0.82 ^c^	1.14 ± 0.06 ^ab^	21.59 ± 1.15 ^a^
H10	20.99 ± 0.80 ^b^	1.03 ± 0.11 ^ab^	20.97 ± 2.10 ^a^
H15	22.81 ± 0.85 ^ab^	0.96 ± 0.06 ^ab^	20.74 ± 1.82 ^a^
H20	24.24 ± 2.95 ^ab^	0.97 ± 0.18 ^ab^	21.90 ± 2.17 ^a^
H25	24.30 ± 3.11 ^ab^	0.96 ± 0.19 ^ab^	21.93 ± 2.04 ^a^
H30	26.60 ± 0.61 ^a^	0.86 ± 0.05 ^b^	22.80 ± 1.28 ^a^

**Table 5:** Sensory characteristics (mean ± standard deviation) of gluten-free sourdough control bread (Ctrl) and gluten-free sourdough breads enriched with 5% (H5), 10% (H10), 15% (H15), 20% (H20), 25% (H25), or 30% (H30) hemp cake meal. Values in the same column with different letters are statistically different (*p* < 0.05) following Fisher’s LSD test.

	Appearance	Texture	Aroma	Taste	Overall
Control	4.45 ± 0.76 ^a^	4.15 ± 0.81 ^a^	4.40 ± 0.88 ^a^	4.20 ± 0.89 ^a^	4.30 ± 0.67 ^a^
H5	4.40 ± 0.60 ^a^	4.10 ± 0.55 ^a^	4.45 ± 0.60 ^a^	4.15 ± 0.59 ^ab^	4.28 ± 0.37 ^a^
H10	4.05 ± 0.76 ^ab^	4.05 ± 0.76 ^a^	4.05 ± 0.69 ^ab^	4.05 ± 0.76 ^ab^	4.05 ± 0.54 ^a^
H15	3.65 ± 0.59 ^bc^	3.55 ± 0.60 ^b^	3.65 ± 0.75 ^bc^	3.70 ± 0.73 ^bc^	3.64 ± 0.36 ^b^
H20	3.30 ± 0.73 ^cd^	3.30 ± 0.73 ^c^	3.35 ± 0.88 ^c^	3.35 ± 0.99 ^c^	3.33 ± 0.69 ^b^
H25	3.00 ± 0.73 ^d^	2.95 ± 0.69 ^c^	2.85 ± 0.67 ^d^	2.65 ± 0.67 ^d^	2.86 ± 0.50 ^c^
H30	2.50 ± 1.00 ^e^	2.30 ± 0.86 ^d^	2.15 ± 0.81 ^e^	1.65 ± 0.67 ^e^	2.15 ± 0.69 ^d^

## Data Availability

The original contributions presented in this study are included in the article/Supplementary Material. Further inquiries can be directed to the corresponding author.

## Supplementary Materials

The following supporting information can be downloaded at https://www.mdpi.com/article/10.3390/foods14203571/s1. Figure S1: Visual texture of rice flour, hemp cake, gluten-free control bread (Ctrl), and gluten-free breads enriched with 5% (H5), 10% (H10), 15% (H15), 20% (H20), 25% (H25), or 30% (H30) hemp cake meal.
