# Supplementary material for: Enhancing the Antioxidant and Nutritional Profile of Gluten-Free Sourdough Bread Using Hemp Press Cake Meal
Source: Foods. 2025 Oct 20;14(20):3571. doi: 10.3390/foods14203571 (PMC12564878; doi:10.3390/foods14203571)
Supplement: Supplementary file 1 [file foods-14-03571-s001.zip › foods-3896697-supplementary.pdf]

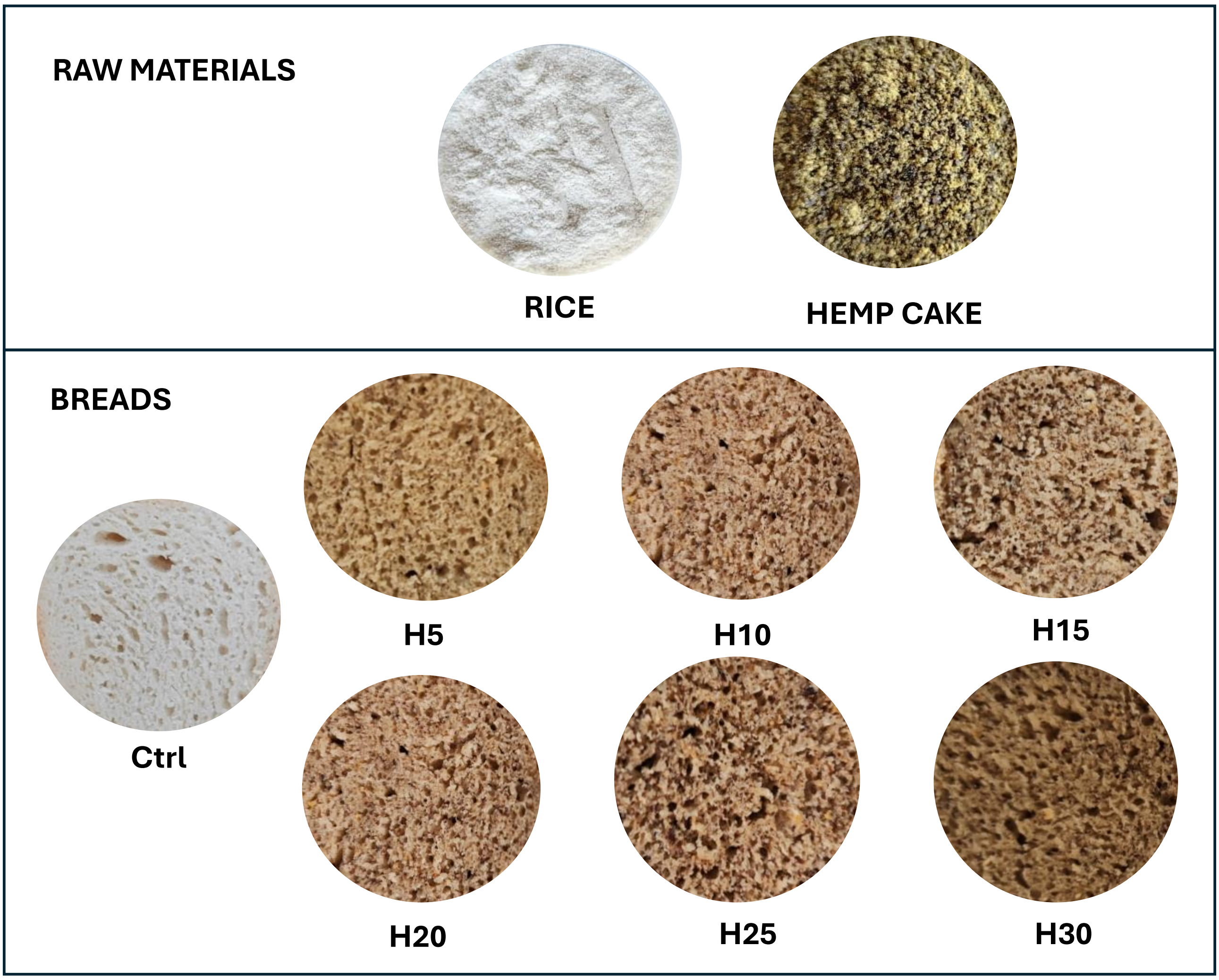

Figure S1. Visual texture of rice flour, hemp cake, gluten-free control bread (Ctrl) and gluten-free breads enriched with 5% (H5), 10% (H10), 15% (H15), 20% (H20), 25% (H25) or 30% (H30) hemp meal.
